# Kindlin‐1 modulates the EGFR pathway and predicts sensitivity to EGFR inhibitors across cancer types

**DOI:** 10.1002/ctm2.813

**Published:** 2022-04-22

**Authors:** Paula Azorin, Florian Bonin, Zakia Tariq, Ambre Petitalot, Florence Coussy, Elisabetta Marangoni, Veronique Becette, Yves Denoux, Anne Vincent‐Salomon, Christophe Le Tourneau, Linda Larbi Cherif, Jerzy Klijanienko, Maud Kamal, Ivan Bièche, Rosette Lidereau, Keltouma Driouch

**Affiliations:** ^1^ Pharmacogenomics Unit, Genetics Department Institut Curie Paris France; ^2^ Department of Medical Oncology Institut Curie Paris France; ^3^ Translational Research Institut Curie, PSL Research University Paris France; ^4^ Pathology Department Institut Curie Paris France; ^5^ Pathology Department Foch Hospital Suresnes France; ^6^ Department of Drug Development and Innovation (D3i) Institut Curie Paris‐Saclay University Paris & Saint Cloud France; ^7^ Present address: Pathology Department, Mignot Hospital, Le Chesnay, France

Dear Editor,

Despite the development of epidermal growth factor receptor (EGFR)‐targeted therapies that have revolutionised the treatment of a broad range of cancers, the efficacy of EGFR inhibitors is limited by the lack of primary response or acquired resistance.^1^, ^2^, ^3^ In this study, we demonstrated that Kindlin‐1 interacts with and regulates the EGFR pathway.^4^, ^5^ We explored the influence of Kindlin‐1 expression in cancer cell lines, patient‐derived xenograft (PDX) models and cohorts of human tumours to reveal its key role in predicting sensitivity to EGFR inhibitors.

Previous works demonstrated that Kindlin‐1 and EGFR interact in keratinocytes.^6^ Thus, we analysed both proteins in a panel of breast cancer cell lines and found that their expression levels were highly correlated (Figure 1A; Figure S1A,B). We next assessed the interaction of Kindlin‐1 and EGFR at the endogenous level in BT20 cells. As shown in Figure 1B, EGFR specifically co‐immunoprecipitated with Kindlin‐1 and reciprocally Kindlin‐1 co‐immunopreciptated with EGFR (Figure 1B). Immunofluorescence experiments revealed the subcellular colocalisation of both proteins. In the absence of epidermal growth factor (EGF), EGFR mainly localised at the plasma membrane, while Kindlin‐1 exhibited dot‐like staining at the perinuclear region of BT20 cells. However, after EGF stimulation, promoting EGFR activation, the receptor was internalised and colocalised with Kindlin‐1 (Figure S1C). Strikingly, the depletion of Kindlin‐1 in these cells resulted in an impaired response to EGF; EGFR remained at the plasma membrane and was no longer internalised (Figure 1C). In addition, Kindlin‐1‐depleted cells showed decreased levels of extracellular signal‐regulated kinase (ERK) and EGFR phosphorylation (1.8‐ and 4.5‐fold change, respectively; Figure 1D; Figure S1D). Conversely, enforced expression of Kindlin‐1 increased ERK phosphorylation (Figure 1E). Thus, in agreement with previous works, our findings suggest that Kindlin‐1 interacts with EGFR, affects its internalisation^6^ and controls EGFR activity in breast cancer cells.

**FIGURE 1:**
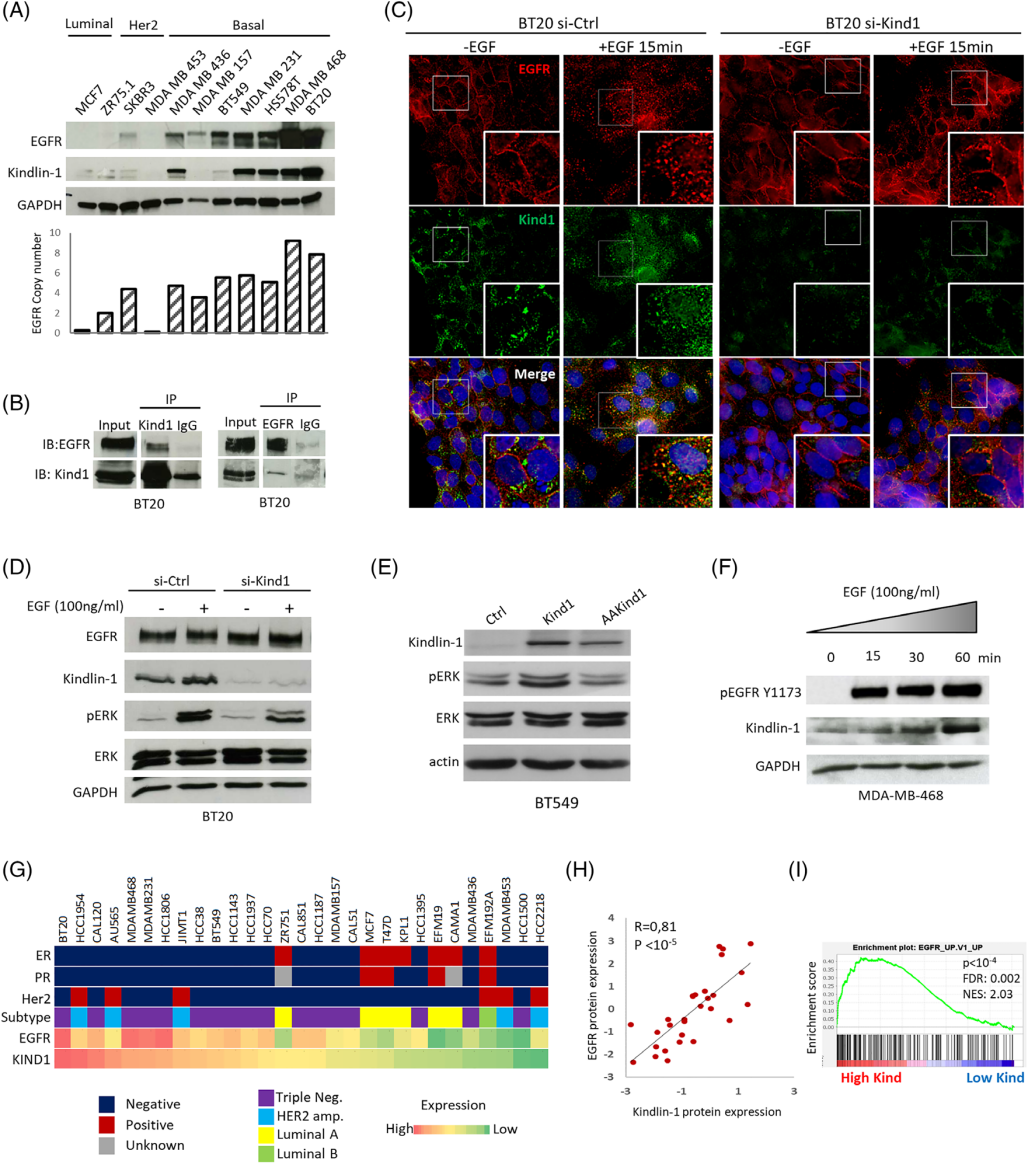
Kindlin‐1 interacts and colocalises with epidermal growth factor receptor (EGFR), promoting the EGFR pathway in breast cancer cells. (A) Western blots of Kindlin‐1 and EGFR in different breast cancer cell lines representing the different subtypes (Luminal, Her2 and Basal) of breast tumours. The EGFR copy number is shown for each cell line. (B) BT20 cells were immunoprecipitated with either normal rabbit IgG, as a negative control (IP: IgG), anti‐Kindlin‐1 (IP: Kind1) or anti‐EGFR (IP: EGFR) antibodies. Immunoprecipitates were examined by immunoblotting with anti‐EGFR and anti‐Kindlin‐1 antibodies. (C) BT20 cells were transfected with control siRNA (si‐Ctrl) or Kindlin‐1 siRNA (si‐Kind1). Seven days after transfection, the cells were starved overnight and then treated or not with 100 ng/ml EGF for 15 min. The cells were then immunostained with anti‐Kindlin‐1 (green) and anti‐EGFR (red) antibodies and counterstained with DAPI (original magnification: X100). (D) Cellular extracts corresponding to (C) were immunoblotted with anti‐EGFR, anti‐Kindlin‐1, anti‐phosphorylated extracellular signal‐regulated kinase (ERK), anti‐ERK and anti‐GAPDH (loading control) antibodies. (E) BT549 cells were stably transfected with wild‐type Kindlin‐1 (Kind1) or the mutant form of Kindlin‐1 deficient in β1‐integrin binding (AAKind; QW611/612AA mutations). Cell extracts were immunoblotted with anti‐Kindlin‐1, anti‐pERK, anti‐ERK and anti‐actin (loading control) antibodies. (F) MDA‐MB‐468 cells were starved overnight and treated with EGF (100 ng/ml) for the indicated times. Cellular extracts were immunoblotted with anti‐pEGFR, anti‐Kindlin‐1 and anti‐GAPDH antibodies. (G) EGFR and Kindlin‐1 protein expression in 29 breast cancer cell lines from CCLE. Estrogen receptor (ER), progesterone receptor (PR), Her2 status and breast cancer subtype have been reported. (H) Correlation between EGFR and Kindlin‐1 protein levels in CCLE cell lines presented in (G) (Spearman's rank correlation test). (I) Gene set enrichment analysis (GSEA) plot showing the enrichment of the EGFR pathway in CCLE cell lines (*n* = 58) categorised into high (*n *= 19) versus low (*n* = 39) Kindlin‐1 expression groups. FDR, false discovery rate q value; NES, normalised enrichment score

Notably, the activation of EGFR signalling increased Kindlin‐1 protein levels, while inhibition of EGFR decreased Kindlin‐1 expression, suggesting that Kindlin‐1 may act as a downstream effector of the EGFR pathway (Figure 1F; Figure S1E).

We next analysed Kindlin‐1 in 58 breast cancer cell lines from CCLE (Figure S2A). Kindlin‐1 and EGFR transcripts were positively correlated (Figure S2B), and the correlation was even stronger at the protein level (Figure 1G‐H). Moreover, a gene set enrichment analysis (GSEA) showed enrichment of several oncogenic pathways associated with EGFR/RAS/MAPK signalling in cells highly expressing Kindlin‐1 (Figures 1I; Figure S2C and Table S1).

To evaluate the clinical relevance of these findings, we examined Kindlin‐1 and EGFR transcripts in breast tumours from The Cancer Genome Atlas (TCGA). Kindlin‐1 transcripts were more abundant in EGFR‐overexpressing tumours (Figure 2A), and these results were corroborated in an independent series of breast cancer patients treated at Institut Curie (Table S2 and Figure S2D). As shown for breast cancer cell lines, the EGFR pathway was significantly enriched in breast cancer patients with high Kindlin‐1 expression (Figure 2B). Furthermore, an immunohistochemical analysis of both proteins in the same patients (*n* = 62) also showed high Kindlin‐1 expression in tumours exhibiting higher levels of EGFR (Figure 2C‐D). Notably, concomitant Kindlin‐1 and EGFR expression was associated with poor patient outcomes; 33% of TCGA triple‐negative breast cancer patients with higher expression of Kindlin‐1 and EGFR showed a lower metastasis‐free survival rate than patients with single overexpression or low expression of both genes (Figure 2E).

**FIGURE 2:**
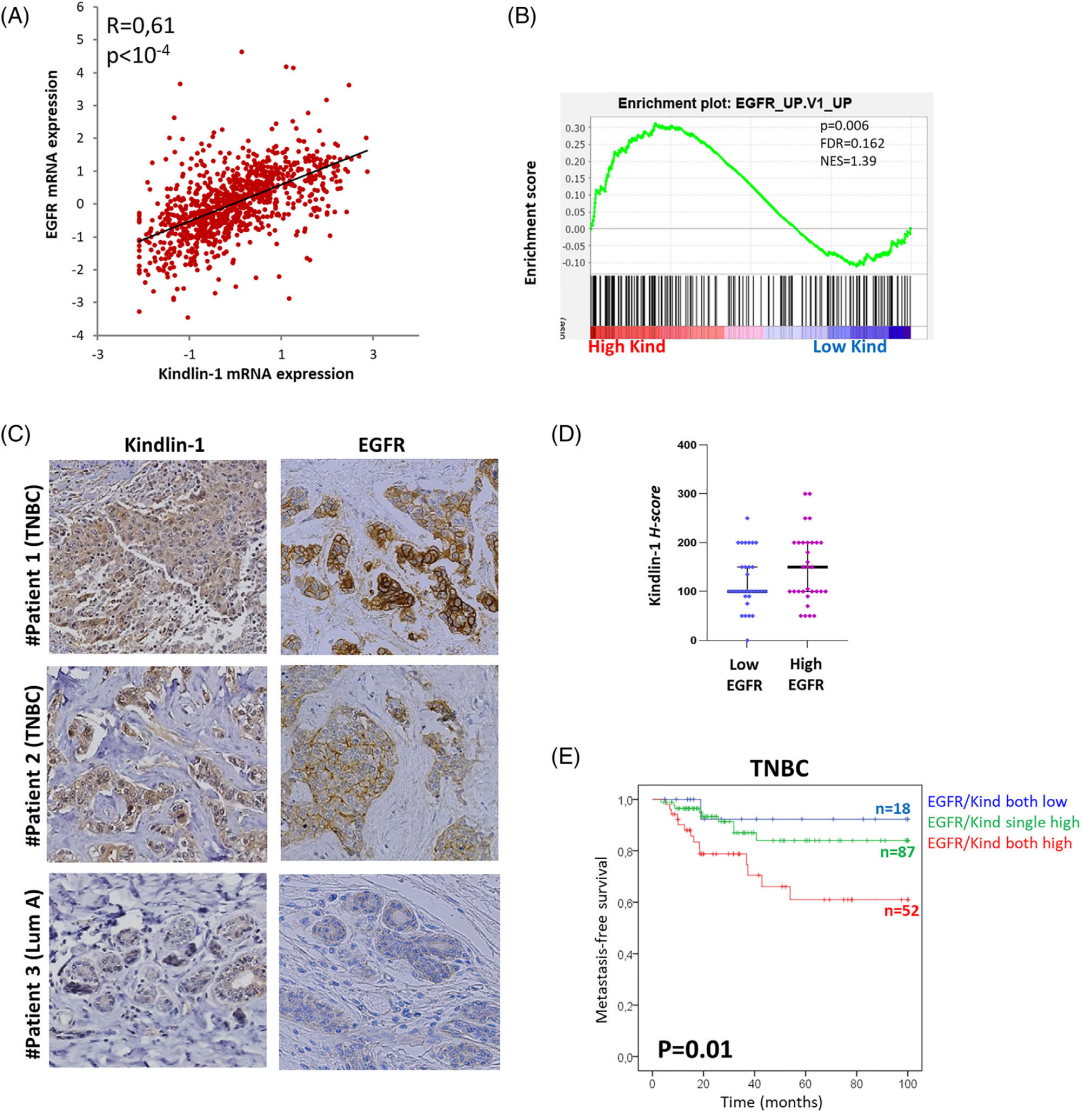
High Kindlin‐1 expression is associated with the epidermal growth factor receptor (EGFR) pathway in breast cancer cell lines and tumours. (A) Correlation between EGFR and Kindlin‐1 mRNA expression levels in 976 breast tumours from The Cancer Genome Atlas (TCGA) (Spearman's rank correlation test). (B) Gene set enrichment analysis (GSEA) plot showing the enrichment of the EGFR pathway in breast tumours from TCGA with high mRNA expression of Kindlin‐1 (*n* = 72/976). C. Kindlin‐1 and EGFR immunohistochemical staining in breast tumours from three different patients with luminal A (Lum A) or triple‐negative breast cancers (TNBCs). These tumours are representative cases of a series of 62 carcinomas from patients treated at the Curie Hospital. (D) Scatter plot showing Kindlin‐1 protein expression in the same series as in (D). Cases were categorised as low versus high EGFR protein expression. Each point represents the H‐score from a single tissue sample ranging from total absence of Kindlin‐1 in the epithelial compartment (H‐score 0) to very strong Kindlin‐1 staining (H‐score 300). (E) Kaplan–Meier curve showing metastasis‐free survival of TNBC patients with respect to EGFR and Kindlin‐1 expression. Patients were divided into three categories: low expression of both EGFR and Kindlin‐1 (blue line); single overexpression of EGFR or Kindlin‐1 (green line); concomitant overexpression of EGFR and Kindlin‐1 (red line) (log‐rank test)

We next examined Kindlin‐1 and EGFR in other EGFR‐driven cancers (TCGA and OncoSG cohorts).^7^ Kindlin‐1 transcripts were increased in carcinomas expressing higher levels of EGFR in head and neck squamous cell carcinoma (HNSCC) and lung and bladder cancers (Figure 3A,E; Figures S3A,B and S4A). GSEA plots demonstrated that the EGFR pathway was enriched in tumours with higher Kindlin‐1 levels (Figure 3B,F; Figure S4B). Immunohistochemical analyses also showed increased Kindlin‐1 expression in those tumours presenting higher levels of EGFR in HNSCC tumours or mutations of EGFR in lung cancers (Figure 3C,G; Figure S5). Furthermore, concomitant Kindlin‐1 and EGFR expression was associated with poor overall survival in HNSCC and lung and bladder cancers (Figure 3D,H; Figures S3C,D and S4C).

**FIGURE 3:**
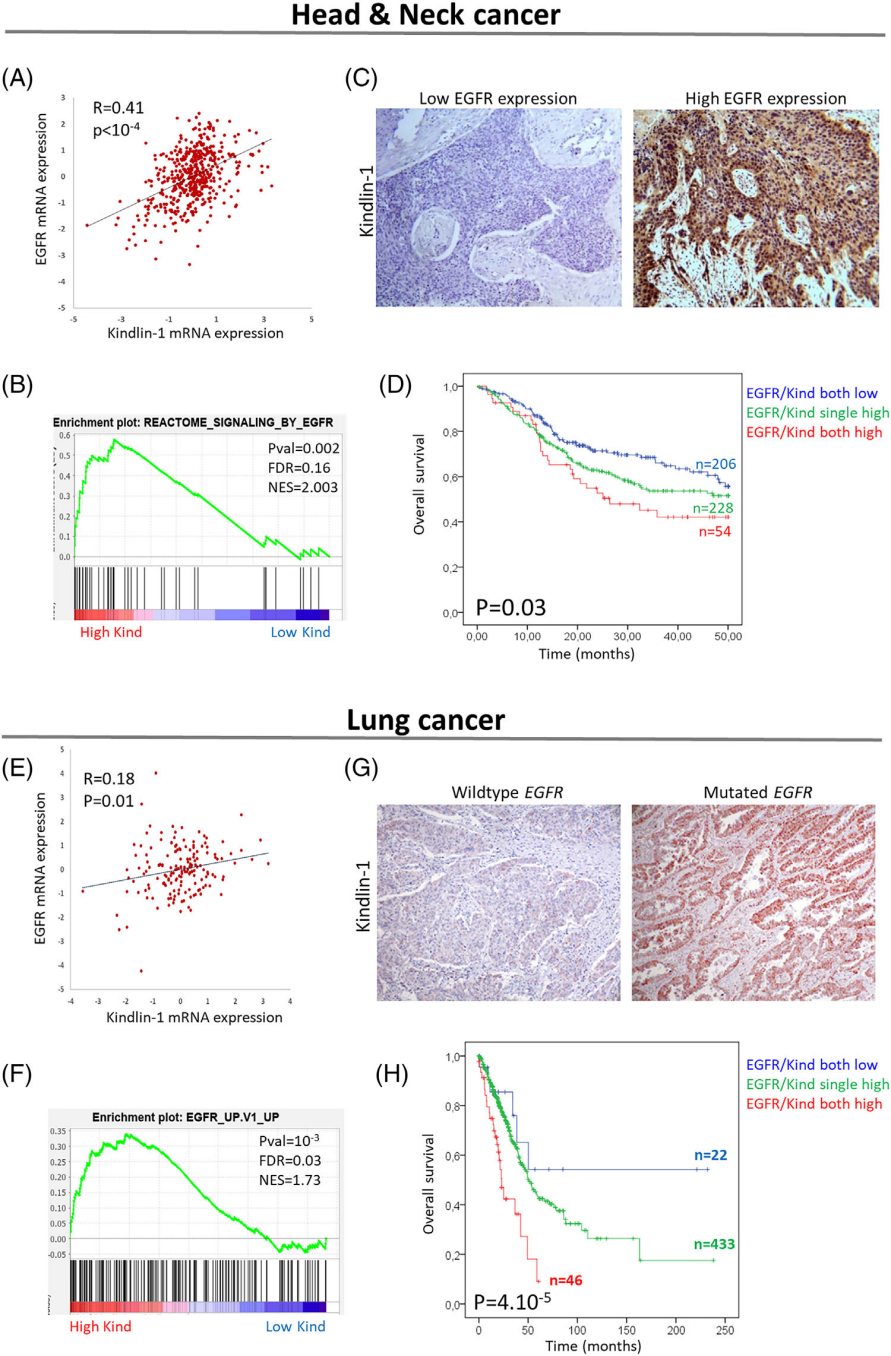
High Kindlin‐1 expression is associated with the epidermal growth factor receptor (EGFR) pathway in head and neck and lung tumors. (A) Correlation between EGFR and Kindlin‐1 mRNA expression levels in head and neck tumours from The Cancer Genome Atlas (TCGA) (*n* = 515) (Spearman's rank correlation test). (B) Gene set enrichment analysis (GSEA) plot showing the enrichment of the EGFR pathway in head and neck tumours with high mRNA expression of Kindlin‐1 (*n* = 64/515). (C) Representative images of Kindlin‐1 immunohistochemical staining in head and neck tumours presenting high or low levels of EGFR. (D) Kaplan–Meier plots showing the overall survival of TCGA head and neck squamous cell carcinoma (HNSCC) with respect to EGFR and Kindlin‐1 expression. (E) Correlation between EGFR and Kindlin‐1 mRNA expression levels in lung tumours from OncoSG (*n* = 169) (Spearman's rank correlation test). (F) GSEA plot showing the enrichment of the EGFR pathway in lung tumours with high mRNA expression of Kindlin‐1 (*n* = 36/169). (G) Representative images of Kindlin‐1 immunohistochemical staining in lung tumours wild‐type for the *EGFR* gene or harbouring an *EGFR* mutation. (H) Kaplan–Meier plots showing the overall survival of TCGA lung cancer patients with respect to EGFR and Kindlin‐1 expression. For Kaplan–Meier analyses, patients were divided into three categories: low expression of both EGFR and Kindlin‐1 (blue line); single overexpression of EGFR or Kindlin‐1 (green line); concomitant overexpression of EGFR and Kindlin‐1 (red line) (log‐rank test)

Hence, we evaluated whether Kindlin‐1 expression was associated with sensitivity to EGFR inhibitors by interrogating the Genomics of Drug Sensitivity in Cancer database. Kindlin‐1 expression was associated with IC50 values for a broad range of EGFR inhibitors in breast, lung, HNSCC and bladder cancer cell lines, suggesting Kindlin‐1 as a predictive biomarker of EGFR‐targeted therapies regardless of epithelial tissue lineage (Figure 4A; Figures S6A and S7). Interestingly, in breast cancer cells, the correlation with the response to cetuximab, a monoclonal antibody widely used in the clinic, was less significant with EGFR (chi square test, *P* = 0.001 vs. 0.02, respectively; Figure 4B). We then used a cohort of 15 PDXs from triple‐negative breast cancers (TNBCs) to test the response to the lapatinib tyrosine kinase inhibitor (Figure 4C). A significant correlation was observed between Kindlin‐1 levels and the tumour growth inhibition induced by lapatinib. The treatment affected PDXs highly expressing Kindlin‐1 but not PDX models with low Kindlin‐1 levels (Figure 4D; Figure S6B).

**FIGURE 4:**
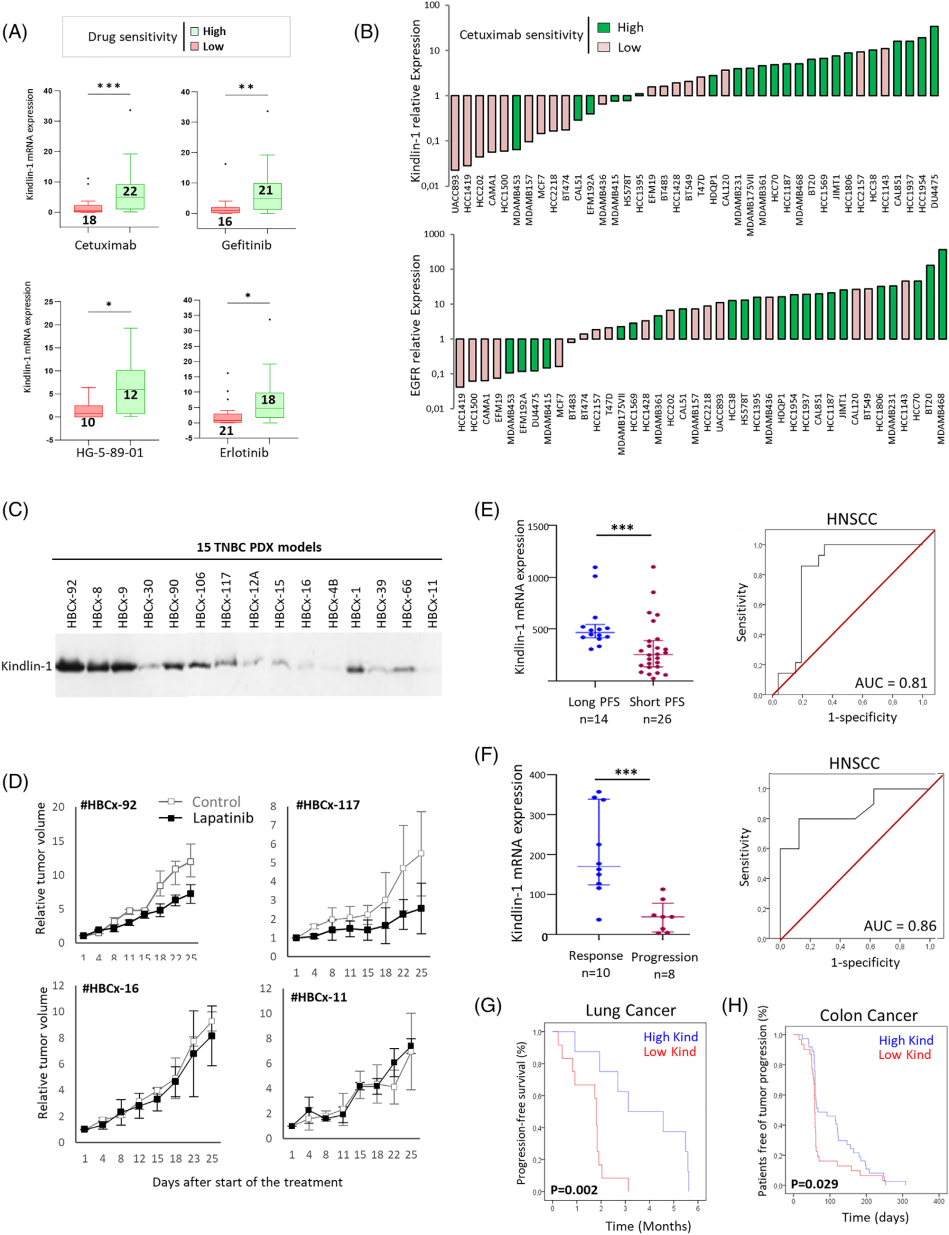
Kindlin‐1 expression is predictive of the response to epidermal growth factor receptor (EGFR) inhibitors. (A) Box and whisker plots representing the differential mRNA expression levels of Kindlin‐1 in breast cancer cells with low versus high sensitivity to cetuximab (*P* = 00009), gefitinib (*P* = 0015), HG‐5‐89‐01 (*P* = 0,02) and erlotinib (*P* = 0013) (Mann–Whitney test). (B) Relative Kindlin‐1 or EGFR expression in 40 breast cancer cell lines from the CCLE classified into high (*n* = 22) or low (*n* = 18) sensitivity to cetuximab depending on their IC50 values as reported in the Genomics of Drug Sensitivity in Cancer database (chi square test, *P* = 0.001 and *P* = 0.02, respectively). (C) Western blot showing Kindlin‐1 protein expression in a series of 15 triple‐negative breast cancer (TNBC) patient‐derived xenograft (PDX) models. (D) Graphs showing relative tumour growth after lapatinib treatment of two PDX models highly expressing Kindlin‐1 (#HBCx‐92 and # HBCx‐117) and two PDX models with low Kindlin‐1 expression (#HBCx‐16 and # HBCx‐11). (E) Scatter plot representing the differential mRNA expression levels of Kindlin‐1 in a dataset of 40 HNSCC patients (GSE65021), dichotomised into long (progression‐free survival [PFS] > 12 months, *n* = 14) and short (PFS < 5.6 months, *n* = 26) PFS after undergoing first‐line CT and cetuximab‐based combination therapy (Mann–Whitney test, *P*  = 0.0008). Receiver operating characteristic (ROC) curve assessing the performance of Kindlin‐1 in the cohort of 40 head and neck squamous cell carcinoma (HNSCC) patients. (F) Scatter plot representing the differential mRNA expression levels of Kindlin‐1 in a series of 18 HNSCC patients from Institut Curie, dichotomised into response (*n* = 10) and progression (*n* = 8) after undergoing cetuximab monotherapy (Mann–Whitney test, *P*  = 0.0008). The ROC curve corresponding to this latter HNSCC cohort resulted in an area under the curve (AUC) of 0.86. (G) Kaplan–Meier plot showing the PFS (%) of 20 non‐small cell lung cancer (NSCLC) cancer patients treated with erlotinib (both *EGFR* and *KRAS* wild‐type) from the BATTLE study (GSE33072, log‐rank test). (H) Kaplan–Meier plot showing the patient‐free survival of tumour progression (%) from a series of 68 metastatic colorectal cancer patients enrolled in a cetuximab monotherapy trial (GSE5851, log‐rank test)

Finally, we analysed a data set of 40 HNSCC patients treated with a first‐line cetuximab‐based combination.^8^ Patients presenting high Kindlin‐1 expression experienced a longer progression‐free survival (PFS) on cetuximab‐based therapy than patients with low expression (median of 468 vs. 254; Figure 4E). These results were validated in an independent series of 18 HNSCC patients treated with cetuximab monotherapy at the Institut Curie. Kindlin‐1 was found to be significantly increased in patients whose tumours were sensitive to cetuximab compared to nonresponder patients (Figure S8A; Figure 4F, median of 170 vs. 46). In both HNSCC cohorts, receiver operating characteristic (ROC) analyses showed significant accuracy in predicting cetuximab outcome, with area under the curve (AUC) values indicating that Kindlin‐1 was strongly associated with disease control (Figure 4E,F). Similar results were obtained in a series of 110 colorectal cancer patients enrolled in a cetuximab monotherapy trial and a series of 20 non‐small cell lung cancer (NSCLC) patients treated with erlotinib.^9^, ^10^ Patients with Kindlin‐1‐overexpressing tumours presented a delayed progression of the disease under EGFR inhibitor treatment compared to patients with low Kindlin‐1 tumours (Figure 4G,H; Figure S8B‐D).

In conclusion, our data support Kindlin‐1 as a promising predictive biomarker of the response to EGFR‐targeted inhibitors. Moreover, our findings provide a rationale for the development of Kindlin‐1‐based therapeutic approaches that may offer new treatment opportunities for a broad range of cancer patients.

## Supporting information

Supporting information.

Supporting information.
**Supplementary Figure 1. Kindlin‐1 colocalizes with EGFR and is associated to EGFR pathway in breast cancer cells. A‐B**. Correlation between EGFR copy number and Kindlin‐1 or EGFR densitometry relative to Figure 1A. **C**. BT20 cells were seeded on fibronectin‐coated coverslips, starved overnight and then treated or not with 100 ng/ml EGF for 15 min. Then, the cells were fixed, permeabilized, immunostained with anti‐Kindlin‐1 (green) and anti‐EGFR (red) antibodies, counterstained with DAPI and imaged with a fluorescence microscope (original magnification: X100). **D**. BT20 cells were transfected with control siRNA (si‐Ctrl) or Kindlin‐1 siRNA (si‐Kind1). Seven days after transfection, cells were starved overnight and then treated or not with 100 ng/ml EGF for 15 min. Cellular extracts were immunoblotted with anti‐EGFR, anti‐pEGFR, anti‐Kindlin‐1 and anti‐GAPDH (loading control) antibodies. **E**. MDA‐MB‐468 and BT20 cells were treated with 10 μM cetuximab for 48 hours. Cellular extracts were immunoblotted with anti‐Kindlin‐1 and anti‐GAPDH antibodies.

Supporting information.
**Supplementary Figure 2. High Kindlin‐1 expression is associated to EGFR pathway in breast cancer. A**. Kindlin‐1 mRNA levels were analyzed for a series of 58 breast cancer cell lines from CCLE. ER, PR Her2, breast cancer subtype as well as EGFR alteration status are reported above Kindlin‐1 expression **B**. Correlation between EGFR and Kindlin‐1 mRNA expression levels in CCLE cell lines (Spearman's rank correlation test) **C**. Heatmap highlighting the top differentially expressed genes in the EGFR gene set. **D**. Correlation between EGFR and Kindlin‐1 mRNA expression levels in breast tumors from the Curie cohort (n = 457) (Spearman's rank correlation test).

Supporting information.
**Supplementary Figure 3. High Kindlin‐1 expression is associated to EGFR pathway in lung cancers**. **A**. Correlation between EGFR and Kindlin‐1 mRNA expression levels in EGFR‐mutated lung tumors from OncoSG (n = 96). **B**. Correlation between EGFR and Kindlin‐1 mRNA expression levels in EGFR‐wildtype lung tumors from OncoSG (n = 73) (Spearman's rank correlation test). **C**. Kaplan‐Meier plot showing overall survival of EGFR‐mutated TCGA lung cancer patients with respect to EGFR and Kindlin‐1 expression. **D**. Kaplan‐Meier plot showing overall survival of EGFR‐wildtype TCGA lung cancer patients with respect to EGFR and Kindlin‐1 expression. C‐D. Patients were divided into three categories: low expression of both EGFR and Kindlin‐1 (blue line); single overexpression of EGFR or Kindlin‐1 (green line); and concomitant overexpression of EGFR and Kindlin‐1 (red line) (Log‐rank test).

Supporting information.
**Supplementary Figure 4. High Kindlin‐1 expression is associated to EGFR pathway in bladder cancers**. **A**. Correlation between EGFR and Kindlin‐1 mRNA expression levels in a series of bladder tumors from the TCGA (n = 407) (Spearman's rank correlation test). **B**. GSEA plot showing the enrichment of EGFR pathway in the same bladder tumors as (A) divided into high and low Kindlin‐1 mRNA levels (n = 131 and n = 276, respectively; FDR: false discovery rate; NES: normalized enrichment score). **C**. Kaplan‐Meier plot showing the overall survival of bladder cancer patients with respect to EGFR and Kindlin‐1 expression. Patients were divided into three categories: low expression of both EGFR and Kindlin‐1 (blue line); single overexpression of EGFR or Kindlin‐1 (green line); concomitant overexpression of EGFR and Kindlin‐1 (red line) (Log‐rank test).

Supporting information.
**Supplementary Figure 5. High Kindlin‐1 expression is associated to EGFR pathway in head and neck cancers**. Representative images of Kindlin‐1 and EGFR immunohistochemical staining in head and neck tumors from patients treated at the Curie Hospital.

Supporting information.
**Supplementary Figure 6. Kindlin‐1 expression is associated with sensitivity to EGFR inhibitors in breast cancer cells**. **A**. Correlation between Kindlin‐1 mRNA expression and reported IC50 values for 16 EGFR inhibitors in the CCLE breast cancer cell lines (n = 44, GDSC database; www.cancerrxgene.org/). Log of p values of the linear regression are shown (*p < 0.05). **B**. Correlation between Kindlin‐1 protein expression and the efficacy of a lapatinib treatment measured as the tumor growth inhibition (TGI) observed in the 15 PDX models (Spearman's rank correlation test).

Supporting information.
**Supplementary Figure 7. Kindlin‐1 expression is associated with the sensitivity to EGFR inhibitors across cancer types**. Box and whisker plots representing the differential mRNA expression levels of Kindlin‐1 in cancer cells with low sensitive versus high sensitivity to different EGFR inhibitors. Statistical analyses were performed using the Mann‐Whitney test (*p < 0.05; **p < 0.01).

Supporting information.
**Supplementary Figure 8. EGFR‐driven cancer patients expressing high levels of Kindlin‐1 exhibit a better survival under EGFR inhibitors A**. Scatter plot representing the differential mRNA expression levels of Kindlin‐1 in normal head and neck tissues (n = 10) vs HNSCC tumors (n = 18) in a series of patients treated at the Curie Hospital (Mann‐Whitney test, p = 0,0002). **B**. Kaplan‐Meier plot showing overall survival under cetuximab treatment in the group of patients with low Kindlin‐1 expression versus and highKindlin‐1 expression (GSE65021, Log‐rank test). **C**. Scatter plot representing the differential mRNA expression levels of Kindlin‐1 in a dataset of 20 NSCLC cancer patients (both *EGFR* and *KRAS* wildtype) from the BATTLE study (GSE33072), dichotomized into long (> 2.5 months, n = 7) and short (< 2.5 months, n = 13) progression‐free survival (PFS) after being treated with erlotinib (Mann‐Whitney test, p = 0.0047). **D**. Scatter plot representing the differential mRNA expression levels of Kindlin‐1 in a dataset of 68 metastatic colorectal cancer patients dichotomized into long (≥ 59 days, n = 43) and short  (< 59 days, n = 25) progression‐free survival (PFS) after enrolling a cetuximab monotherapy trial (GSE5851, Mann‐Whitney test, p = 0.0052).

Supporting information.
**Supplementary Table 1**. Clinical data from breast cancer patients treated at Institut Curie.

Supporting information.
**Supplementary Table 2**. Signaling pathways enriched in breast cancer cell lines with high Kindlin‐1 mRNA expression levels as obtained by performing a Gene Set Enrichment Analysis (GSEA). EGFR/RAS/MAPK‐related pathways are highlighted in red. ES: enrichment score; NES: normalized enrichment score; FDR: false discovery rate.

Supporting information.
