# Supplementary material for: Kindlin‐1 modulates the EGFR pathway and predicts sensitivity to EGFR inhibitors across cancer types
Source: Clin Transl Med. 2022 Apr 22;12(4):e813. doi: 10.1002/ctm2.813 (PMC9029018; doi:10.1002/ctm2.813)
Supplement: Supplementary file 1 — Supporting information. [file CTM2-12-e813-s008.docx]

**Supplementary Figure 1. Kindlin-1 colocalizes with EGFR and is associated to EGFR pathway in breast cancer cells. A-B.** Correlation between EGFR copy number and Kindlin-1 or EGFR densitometry relative to Figure 1A. **C.** BT20 cells were seeded on fibronectin-coated coverslips, starved overnight and then treated or not with 100ng/ml EGF during 15min. Then, cells were fixed, permeabilized, immunostained with anti-Kindlin-1 (green) and anti-EGFR (red) antibodies, counterstained with DAPI and imaged with a fluorescence microscope (original magnification: X100). **D.** BT20 cells were transfected with control siRNA (si-Ctrl) or Kindlin-1 siRNA (si-Kind1). Seven days after transfection, cells were starved overnight and then treated or not with 100ng/ml EGF during 15min. Cellular extracts were immunoblotted with anti-EGFR, anti-pEGFR, anti-Kindlin-1 and anti-GAPDH (loading control) antibodies. **E.** MDA-MB-468 and BT20 cells were treated with 10µM cetuximab for 48 hours. Cellular extracts were immunoblotted with anti-Kindlin-1 and anti-GAPDH antibodies.

**Supplementary Figure 2. High Kindlin-1 expression is associated to EGFR pathway in breast cancer. A.** Kindlin-1 mRNA levels were analyzed for a series of 58 breast cancer cell lines from CCLE. ER, PR Her2, breast cancer subtype as well as EGFR alteration status are reported above Kindlin-1 expression **B.** Correlation between EGFR and Kindlin-1 mRNA expression levels in CCLE cell lines (Spearman’s rank correlation test) **C.** Heatmap highlighting the top differentially expressed genes in the EGFR gene set. **D.** Correlation between EGFR and Kindlin-1 mRNA expression levels in breast tumors from the Curie cohort (n=457) (Spearman’s rank correlation test).

**Supplementary Figure 3.** **High Kindlin-1 expression is associated to EGFR pathway in lung cancers**. **A.** Correlation between EGFR and Kindlin-1 mRNA expression levels in EGFR-mutated lung tumors from OncoSG (n=96). **B.** Correlation between EGFR and Kindlin-1 mRNA expression levels in EGFR-wildtype lung tumors from OncoSG (n=73) (Spearman’s rank correlation test). **C.** Kaplan-Meier plot showing overall survival of EGFR-mutated TCGA lung cancer patients with respect to EGFR and Kindlin-1 expression. **D.** Kaplan-Meier plot showing overall survival of EGFR-wildtype TCGA lung cancer patients with respect to EGFR and Kindlin-1 expression. C-D. Patients were divided into three categories: low expression of both EGFR and Kindlin-1 (blue line); single overexpression of EGFR or Kindlin-1 (green line); concomitant overexpression of EGFR and Kindlin-1 (red line) (Log-rank test).

**Supplementary Figure 4.** **High Kindlin-1 expression is associated to EGFR pathway in bladder cancers**. **A.** Correlation between EGFR and Kindlin-1 mRNA expression levels in a series of bladder tumors from the TCGA (n=407) (Spearman’s rank correlation test). **B.** GSEA plot showing the enrichment of EGFR pathway in the same bladder tumors as (A) divided into high and low Kindlin-1 mRNA levels (n=131 and n=276, respectively; FDR: false discovery rate; NES: normalized enrichment score). **C.** Kaplan-Meier plot showing overall survival of bladder cancer patients with respect to EGFR and Kindlin-1 expression. Patients were divided into three categories: low expression of both EGFR and Kindlin-1 (blue line); single overexpression of EGFR or Kindlin-1 (green line); concomitant overexpression of EGFR and Kindlin-1 (red line) (Log-rank test).

**Supplementary Figure 5.** **High Kindlin-1 expression is associated to EGFR pathway in head and neck cancers**. Representative images of Kindlin-1 and EGFR immunohistochemical staining in head and neck tumors from patients treated at the Curie Hospital

**Supplementary Figure 6.** **Kindlin-1 expression is associated with sensitivity to EGFR inhibitors in breast cancer cells**. **A.** Correlation between Kindlin-1 mRNA expression and reported IC50 values for 16 EGFR inhibitors in the CCLE breast cancer cell lines (n=44, GDSC database; www.cancerrxgene.org/). Log of p-values of the linear regression are shown (*p < 0.05). **B.** Correlation between Kindlin-1 protein expression and the efficacy of a lapatinib treatment measured as the tumor growth inhibition (TGI) observed in the 15 PDX models (Spearman’s rank correlation test).

**Supplementary Figure 7.** **Kindlin-1 expression is associated with the sensitivity to EGFR inhibitors across cancer types.** Box and whisker plots representing the differential mRNA expression levels of Kindlin-1 in cancer cells with low sensitive versus high sensitivity to different EGFR inhibitors. Statistical analyses were performed using the Mann-Whitney test (*p < 0.05; **p < 0.01).

**Supplementary Figure 8. EGFR-driven cancer patients expressing high levels of Kindlin-1 exhibit a better survival under EGFR inhibitors A.** Scatter plot representing the differential mRNA expression levels of Kindlin-1 in normal head and neck tissues (n=10) vs HNSCC tumors (n=18) in a series of patients treated at the Curie Hospital (Mann-Whitney test, p=0,0002). **B.** Kaplan-Meier plot showing overall survival under cetuximab treatment in the group of patients with low Kindlin-1 expression versus and highKindlin-1 expression (GSE65021, Log-rank test). **C.** Scatter plot representing the differential mRNA expression levels of Kindlin-1 in a dataset of 20 NSCLC cancer patients (both *EGFR* and *KRAS* wildtype) from the BATTLE study (GSE33072), dichotomized into long (> 2.5 months, n=7) and short (<2.5 months, n=13) progression-free survival (PFS) after being treated with erlotinib (Mann-Whitney test, p=0.0047). **D.** Scatter plot representing the differential mRNA expression levels of Kindlin-1 in a dataset of 68 metastatic colorectal cancer patients dichotomized into long (≥ 59 days, n=43) and short (< 59 days, n=25) progression-free survival (PFS) after enrolling a cetuximab monotherapy trial (GSE5851, Mann-Whitney test, p=0.0052).

**Supplementary Table 1.** Clinical data from breast cancer patients treated at Institut Curie.

**Supplementary Table 2.** Signaling pathways enriched in breast cancer cell lines with high Kindlin-1 mRNA expression levels as obtained by performing a Gene Set Enrichment Analysis (GSEA). EGFR/RAS/MAPK-related pathways are highlighted in red. ES: enrichment score; NES: normalized enrichment score; FDR: false discovery rate.
