# Supplementary figures and images for: Kindlin‐1 modulates the EGFR pathway and predicts sensitivity to EGFR inhibitors across cancer types
Source: Clin Transl Med. 2022 Apr 22;12(4):e813. doi: 10.1002/ctm2.813 (PMC9029018; doi:10.1002/ctm2.813)

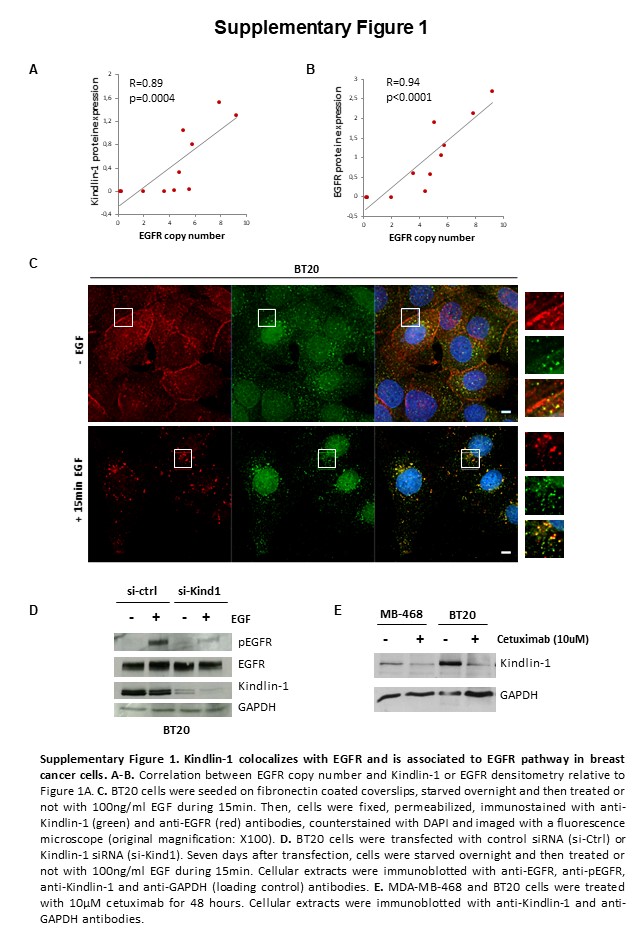

Supplement: Supplementary file 2 — Supporting information. Supplementary Figure 1. Kindlin‐1 colocalizes with EGFR and is associated to EGFR pathway in breast cancer cells. A‐B. Correlation between EGFR copy number and Kindlin‐1 or EGFR densitometry relative to Figure 1A. C. BT20 cells were seeded on fibronectin‐coated coverslips, starved overnight and then treated or not with 100 ng/ml EGF for 15 min. Then, the cells were fixed, permeabilized, immunostained with anti‐Kindlin‐1 (green) and anti‐EGFR (red) antibodies, counterstained with DAPI and imaged with a fluorescence microscope (original magnification: X100). D. BT20 cells were transfected with control siRNA (si‐Ctrl) or Kindlin‐1 siRNA (si‐Kind1). Seven days after transfection, cells were starved overnight and then treated or not with 100 ng/ml EGF for 15 min. Cellular extracts were immunoblotted with anti‐EGFR, anti‐pEGFR, anti‐Kindlin‐1 and anti‐GAPDH (loading control) antibodies. E. MDA‐MB‐468 and BT20 cells were treated with 10 μM cetuximab for 48 hours. Cellular extracts were immunoblotted with anti‐Kindlin‐1 and anti‐GAPDH antibodies. [file CTM2-12-e813-s009.JPG]

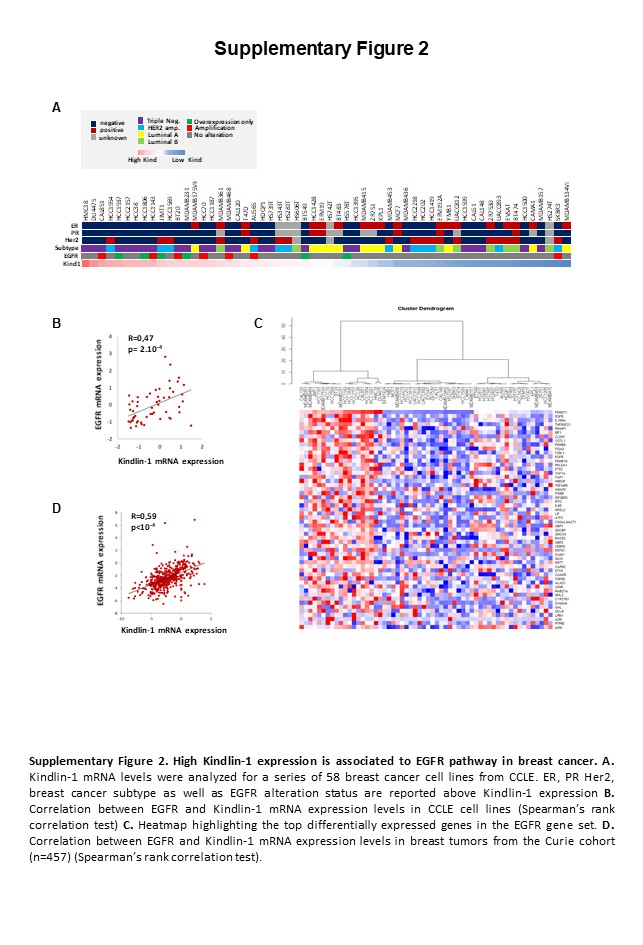

Supplement: Supplementary file 3 — Supporting information. Supplementary Figure 2. High Kindlin‐1 expression is associated to EGFR pathway in breast cancer. A. Kindlin‐1 mRNA levels were analyzed for a series of 58 breast cancer cell lines from CCLE. ER, PR Her2, breast cancer subtype as well as EGFR alteration status are reported above Kindlin‐1 expression B. Correlation between EGFR and Kindlin‐1 mRNA expression levels in CCLE cell lines (Spearman's rank correlation test) C. Heatmap highlighting the top differentially expressed genes in the EGFR gene set. D. Correlation between EGFR and Kindlin‐1 mRNA expression levels in breast tumors from the Curie cohort (n = 457) (Spearman's rank correlation test). [file CTM2-12-e813-s003.JPG]

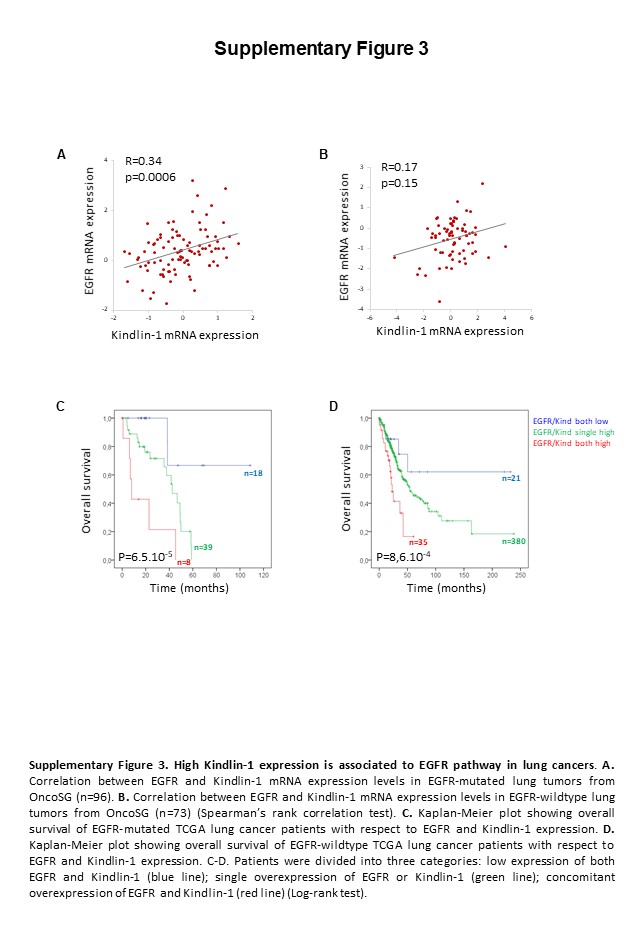

Supplement: Supplementary file 4 — Supporting information. Supplementary Figure 3. High Kindlin‐1 expression is associated to EGFR pathway in lung cancers. A. Correlation between EGFR and Kindlin‐1 mRNA expression levels in EGFR‐mutated lung tumors from OncoSG (n = 96). B. Correlation between EGFR and Kindlin‐1 mRNA expression levels in EGFR‐wildtype lung tumors from OncoSG (n = 73) (Spearman's rank correlation test). C. Kaplan‐Meier plot showing overall survival of EGFR‐mutated TCGA lung cancer patients with respect to EGFR and Kindlin‐1 expression. D. Kaplan‐Meier plot showing overall survival of EGFR‐wildtype TCGA lung cancer patients with respect to EGFR and Kindlin‐1 expression. C‐D. Patients were divided into three categories: low expression of both EGFR and Kindlin‐1 (blue line); single overexpression of EGFR or Kindlin‐1 (green line); and concomitant overexpression of EGFR and Kindlin‐1 (red line) (Log‐rank test). [file CTM2-12-e813-s001.JPG]

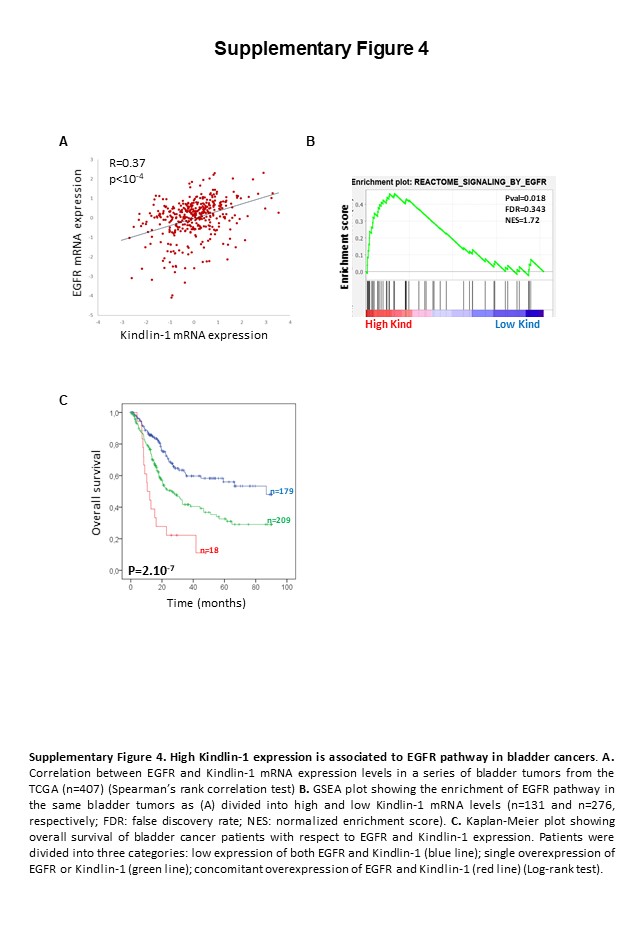

Supplement: Supplementary file 5 — Supporting information. Supplementary Figure 4. High Kindlin‐1 expression is associated to EGFR pathway in bladder cancers. A. Correlation between EGFR and Kindlin‐1 mRNA expression levels in a series of bladder tumors from the TCGA (n = 407) (Spearman's rank correlation test). B. GSEA plot showing the enrichment of EGFR pathway in the same bladder tumors as (A) divided into high and low Kindlin‐1 mRNA levels (n = 131 and n = 276, respectively; FDR: false discovery rate; NES: normalized enrichment score). C. Kaplan‐Meier plot showing the overall survival of bladder cancer patients with respect to EGFR and Kindlin‐1 expression. Patients were divided into three categories: low expression of both EGFR and Kindlin‐1 (blue line); single overexpression of EGFR or Kindlin‐1 (green line); concomitant overexpression of EGFR and Kindlin‐1 (red line) (Log‐rank test). [file CTM2-12-e813-s002.JPG]

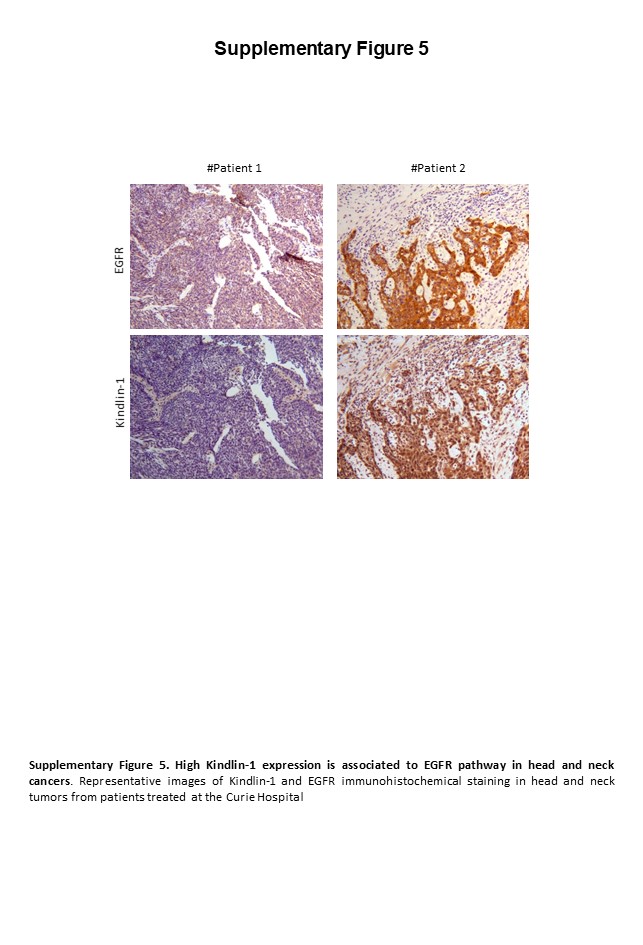

Supplement: Supplementary file 6 — Supporting information. Supplementary Figure 5. High Kindlin‐1 expression is associated to EGFR pathway in head and neck cancers. Representative images of Kindlin‐1 and EGFR immunohistochemical staining in head and neck tumors from patients treated at the Curie Hospital. [file CTM2-12-e813-s012.JPG]

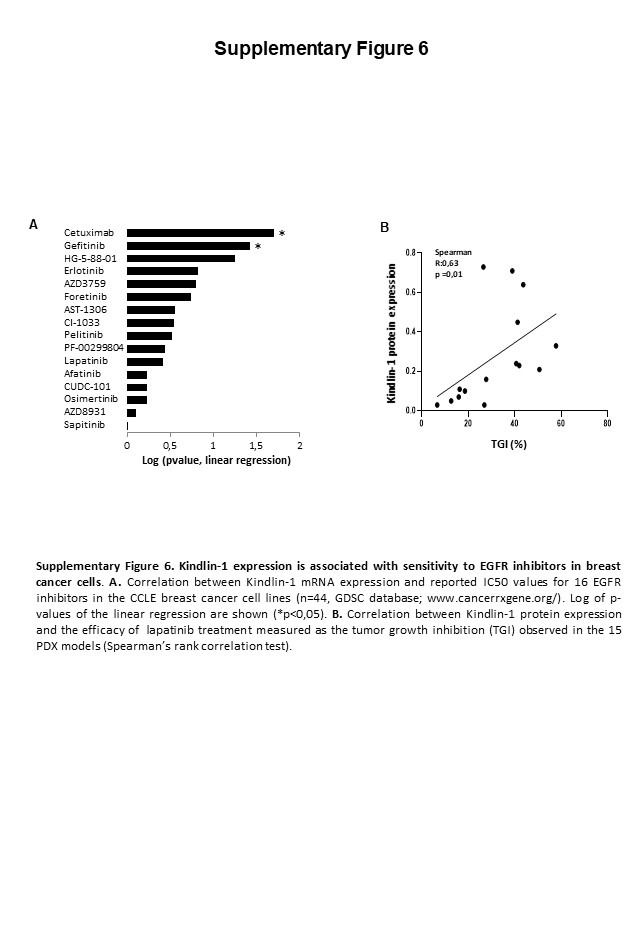

Supplement: Supplementary file 7 — Supporting information. Supplementary Figure 6. Kindlin‐1 expression is associated with sensitivity to EGFR inhibitors in breast cancer cells. A. Correlation between Kindlin‐1 mRNA expression and reported IC50 values for 16 EGFR inhibitors in the CCLE breast cancer cell lines (n = 44, GDSC database; www.cancerrxgene.org/). Log of p values of the linear regression are shown (*p < 0.05). B. Correlation between Kindlin‐1 protein expression and the efficacy of a lapatinib treatment measured as the tumor growth inhibition (TGI) observed in the 15 PDX models (Spearman's rank correlation test). [file CTM2-12-e813-s007.JPG]

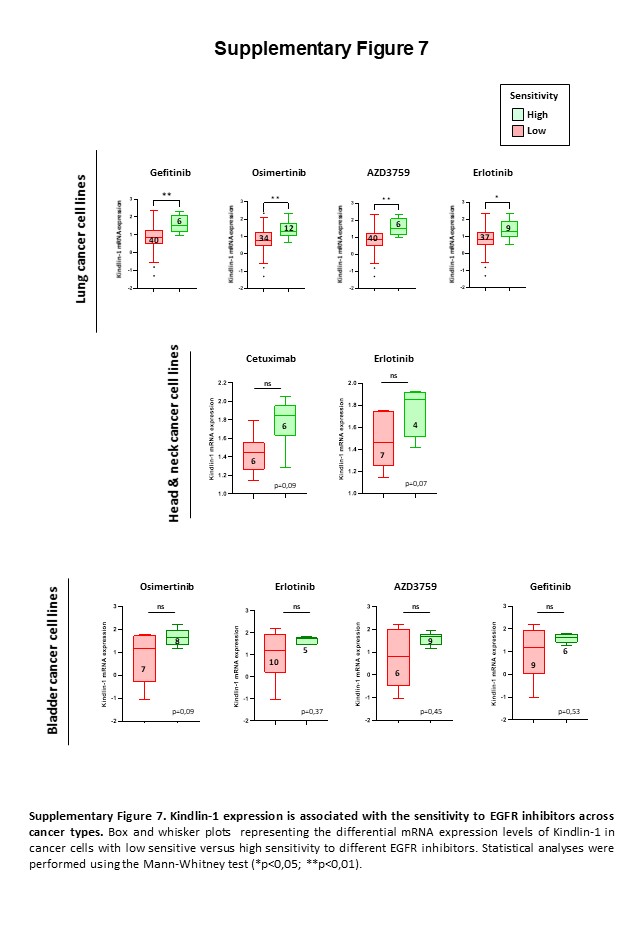

Supplement: Supplementary file 8 — Supporting information. Supplementary Figure 7. Kindlin‐1 expression is associated with the sensitivity to EGFR inhibitors across cancer types. Box and whisker plots representing the differential mRNA expression levels of Kindlin‐1 in cancer cells with low sensitive versus high sensitivity to different EGFR inhibitors. Statistical analyses were performed using the Mann‐Whitney test (*p < 0.05; **p < 0.01). [file CTM2-12-e813-s005.JPG]

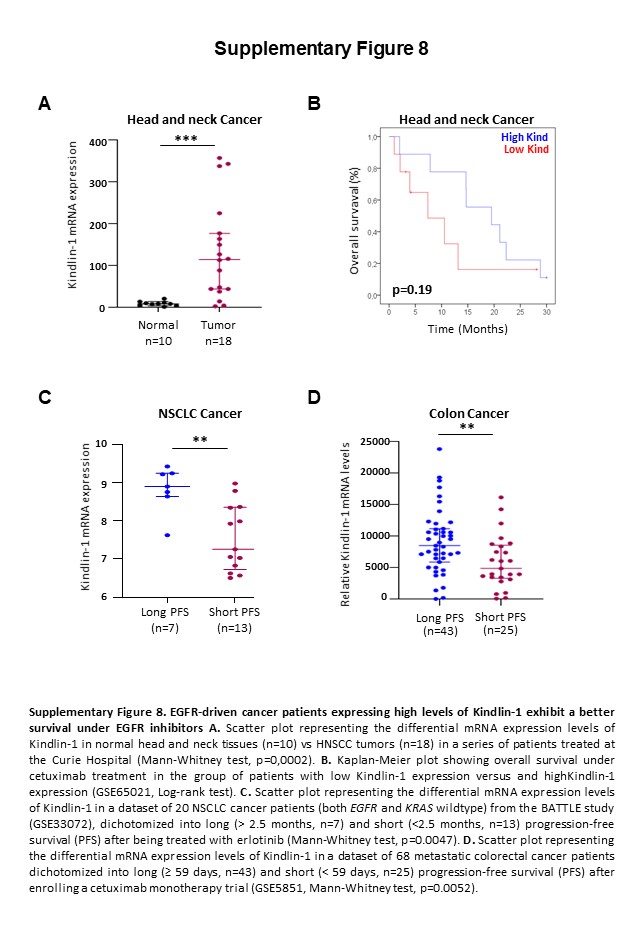

Supplement: Supplementary file 9 — Supporting information. Supplementary Figure 8. EGFR‐driven cancer patients expressing high levels of Kindlin‐1 exhibit a better survival under EGFR inhibitors A. Scatter plot representing the differential mRNA expression levels of Kindlin‐1 in normal head and neck tissues (n = 10) vs HNSCC tumors (n = 18) in a series of patients treated at the Curie Hospital (Mann‐Whitney test, p = 0,0002). B. Kaplan‐Meier plot showing overall survival under cetuximab treatment in the group of patients with low Kindlin‐1 expression versus and highKindlin‐1 expression (GSE65021, Log‐rank test). C. Scatter plot representing the differential mRNA expression levels of Kindlin‐1 in a dataset of 20 NSCLC cancer patients (both EGFR and KRAS wildtype) from the BATTLE study (GSE33072), dichotomized into long (> 2.5 months, n = 7) and short (< 2.5 months, n = 13) progression‐free survival (PFS) after being treated with erlotinib (Mann‐Whitney test, p = 0.0047). D. Scatter plot representing the differential mRNA expression levels of Kindlin‐1 in a dataset of 68 metastatic colorectal cancer patients dichotomized into long (≥ 59 days, n = 43) and short (< 59 days, n = 25) progression‐free survival (PFS) after enrolling a cetuximab monotherapy trial (GSE5851, Mann‐Whitney test, p = 0.0052). [file CTM2-12-e813-s004.JPG]

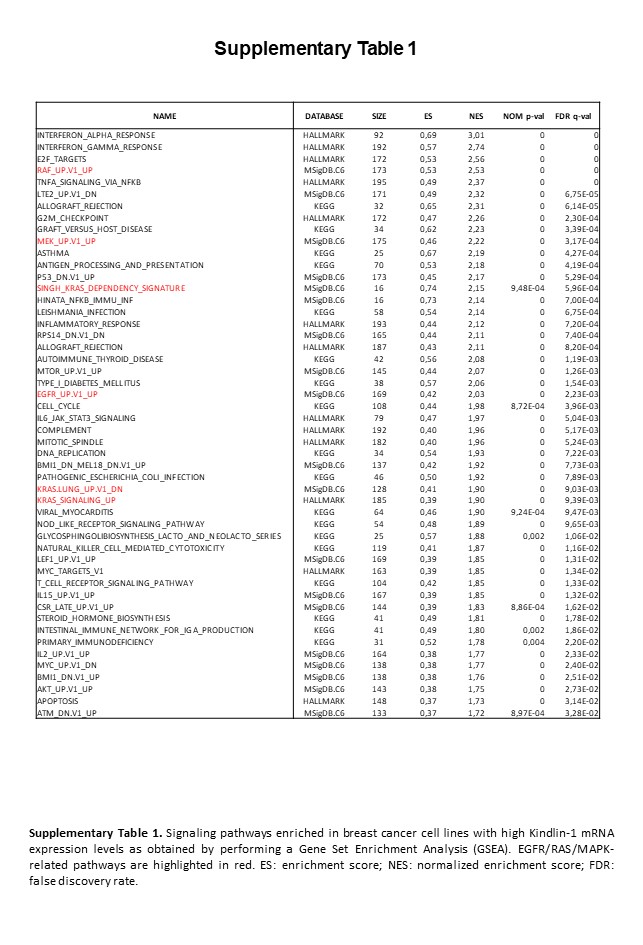

Supplement: Supplementary file 10 — Supporting information. Supplementary Table 1. Clinical data from breast cancer patients treated at Institut Curie. [file CTM2-12-e813-s006.JPG]

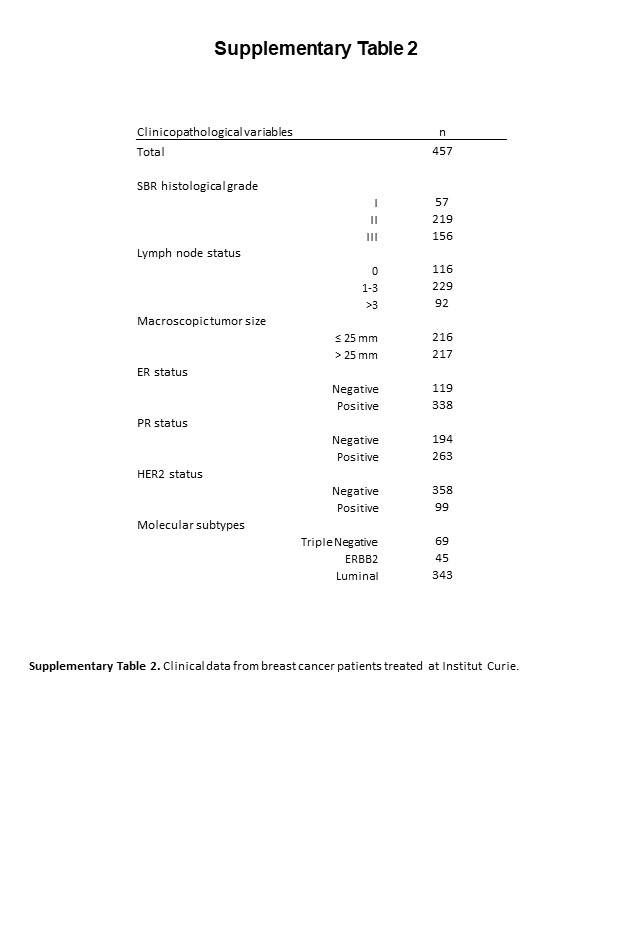

Supplement: Supplementary file 11 — Supporting information. Supplementary Table 2. Signaling pathways enriched in breast cancer cell lines with high Kindlin‐1 mRNA expression levels as obtained by performing a Gene Set Enrichment Analysis (GSEA). EGFR/RAS/MAPK‐related pathways are highlighted in red. ES: enrichment score; NES: normalized enrichment score; FDR: false discovery rate. [file CTM2-12-e813-s011.JPG]
