# Supplementary material for: Kindlin‐1 modulates the EGFR pathway and predicts sensitivity to EGFR inhibitors across cancer types
Source: Clin Transl Med. 2022 Apr 22;12(4):e813. doi: 10.1002/ctm2.813 (PMC9029018; doi:10.1002/ctm2.813)
Supplement: Supplementary file 12 — Supporting information. [file CTM2-12-e813-s010.docx]

**MATERIAL AAND METHODS**

***Cell culture***

Human breast cancer cell lines MCF7, ZR75.1, SKBR3, MDA-MB-453, MDA-MB-436, MDA-MB-157, BT549, MDA-MB-231, HS578T, MDA-MB-468 and BT20 were purchased from ATCC (Manassas,VA,USA). Cells were grown in DMEM, RPMI 1640, MEM Alpha or Leibovitz's L-15 medium supplemented with 10% FBS and 1% antibiotics (50 µg/mL penicillin, 50 µg/mL streptomycin, 100 µg/mL neomycin) and maintained at 37°C with 5% CO_2_ (without CO_2_ for Leibovitz's L-15 medium).

***Knockdown experiments***

Transfections were performed using JetPrime (Polyplus-transfection, New York, NY, USA) following the manufacturer’s protocol with siRNA-negative control (D-001210-03) or siRNA-Kindlin-1 (D-004511-02) from Dharmacon (Lafayette, CO, USA) for transient silencing experiments.

***Expression constructs and transfections***

The human *KIND1* cDNA was subcloned into pIREShyg3 vectors as previously described (31). The Kindlin-1 PTB mutation QW611/612AA was introduced with a site-directed mutagenesis using the QuikChange site-directed mutagenesis kit (Agilent, Santa Clara, CA), following the manufacturers recommendations. The primers used for mutagenesis were F5'-CATGGAGATTCACAAATATCAAAGCGGCGAATGTAAACTGGGAAACCCGGC-3’ and R3’-GTACCTCTAAGTGTTTATAGTTTCGCCGCTTACATTTGACCCTTTGGGCCG-5’. Transfections were performed using Lipofectamine (Invitrogen) following the manufacturer’s instructions. Stable transfectants were grown in the presence of 200µg/mL hygromycin (Sigma-Aldrich, St Louis, MO).

***Western blotting***

Cells were lysed using RIPA buffer (50 mM Tris-HCl, pH 8; 150 mM NaCl; 0.5% triton; 0.5% deoxycholic acid) containing protease inhibitors (1:1000 orthovanadate, 1:1000 apoprotinine, 1:200 PMSF). Protein extracts were loaded on a polyacrylamide gel, transferred to a nitrocellulose membrane and incubated overnight at 4° C with primary antibodies for EGFR (1:1000, 4267S), pEGFR (1:1000, 4407S), pERK1/2 (1:1000, 4370S), ERK1/2 (1:1000, 9102S), GAPDH (1:1000, 3683S), all purchased from Cell Signaling (Danvers, MA), or Kindlin-1 (1:20000,(31). The signals were detected according to the ECL Western Blotting Analysis System procedure (GE Healthcare, Buckinghamshire, UK).

***Co-immunoprecipitation***

BT-20 cells were grown in complete media, supplemented with 10% FBS. Cells were then lysed using NP40 buffer (50 mM Tris-HCl, pH 7.5; 150 mM NaCl; 0.5% NP40) containing protease inhibitors (1:1000 orthovanadate, 1:1000 apoprotinine, 1:200 PMSF). Protein extracts were incubated with 1μg antibodies for Kindlin-1, EGFR or normal rabbit IgG (GTX35035, GeneTex, Irvine, CA, USA) and 10 μl Sepharose Protein A beads (Rockland, Limerick, PA, USA) at 4°C overnight. Beads were washed with NP40 buffer three times and immunoprecipitates were resolved by western blotting.

***Immunofluorescence***

Cells were plated on fibronectin coated coverslips. After serum starvation overnight, cells were treated with 100ng/ml EGF for the indicated time, fixed in 4% paraformaldehyde, permeabilized and immunostained with primary antibodies (Kindlin-1, 1:700 or EGFR, 1:250) followed by alexa fluor-conjugated secondary antibodies (A11031 and A11034, Invitrogen, Carlsbad, CA, USA). Cells were then counterstained with DAPI and imaged with the fluorescence Eclipse Ti microscope from Nikon (Melville, NY, USA).

***Gene expression analysis***

Microarray expression for cancer cell lines (58 breast, 174 lung, 30 head and neck and 24 bladder) from the Cancer Cell Line Encyclopedia (CCLE) and RNA-Seq of tumors from the TCGA project (1082 breast, 510 lung, 515 head and neck and 407 bladder), and 169 lung tumors from the OncoSG dataset, were publicly available from cBioPortal ([www.cbioportal.org/](http://www.cbioportal.org/)).

Gene expressions were analyzed by GSEA v4.0.3 software (33) using gene sets obtained from the Molecular Signatures Database (MSigDB 7.2; <http://software.broadinstitute.org/gsea/msigdb/>). High versus low Kindlin-1 expression groups were categorized by applying the optimal cutoff point determined by a hierarchical clustering on Kindlin-1 expression.

***Kindlin-1 expression analysis in human breast, lung, head and neck tumors***

We performed qRT-PCR analysis of a series of 457 primary breast tumor samples collected from patients undergoing surgery at the Institut Curie (Supplementary Table S2), and a series of head and neck squamous cell carcinomas from 18 patients undergoing cetuximab monotherapy at the Institut Curie.

For the immunohistochemistry analyses, a tissue microarrays (TMAs) consisting of 62 breast tumors and adjacent normal breast tissues from patients treated at the Institut Curie was obtained from the Pathology Department. In addition, a series of 96 human lung adenocarcinomas for which EGFR mutational status had been determined and a prospective series of 10 human head and neck squamous cell carcinomas (SCANDARE trial, NCT03017573) were obtained from Foch Hospital and Institut Curie, respectively.

***Immunohistochemistry***

Tumor sections were deparaffinized in toluene, rehydrated in ethanol and water, immersed in Tris-EDTA recovery buffer (10 mM Tris-base, 1mM EDTA, 0.05% Tween 20, pH9) and treated with peroxidase blocking reagent (Dako, Ely, UK). For breast and lung tumors, sections were incubated at 4°C overnight with anti-Kindlin-1 (1:500 AB68041, Abcam, Cambridge, MA) or EGFR (clone D38B1, 1:50, 4267 Cell signaling) antibodies. Staining signals were then revealed with the Dako REAL detection system, Peroxidase/AEC kit (Dako, Ely, UK). For head and neck tumors, sections were incubated with anti-Kindlin (Clone D1K4C, 1:600, 36734S, cell Signaling) or EGFR (clone D38B1, 1:50) antibodies and the signals were revealed with DAB. The slides were counterstained with Mayer's hematoxylin. For semiquantitative analysis, the H-score method assigned a score from 0 to 300 to each patient, based on the percentage of cells stained at different intensities.

***Breast cancer patient derived xenografts (PDX)***

Fifteen triple negative breast cancer PDX models were obtained as previously described (34). Informed consent was obtained from patients before xenograft establishment. When tumors reached a volume of 60 to 200 mm^3^, mice were randomly assigned to the control or treated groups. Lapatinib, purchased from GSK, was administrated orally at a dose of 200mg/kg, 5 times a week. The vehicle alone, MCT (0.5% methylcellulose and 0.2% tween80) was similarly administered to 4 mice of the control group.

Tumor growth inhibition (TGI) was calculated using the following formula [1-(V_ft_-V_0t_)/(V_fc_-V_0c_))]*100 where V_ft_ = final volume of the treated group (at the end of the treatment); V_0t_ = initial volume of the treatment group (at the beginning of the treatment); V_fc_ = final volume of the control group (at the end of the treatment); V_0c_ = initial volume of the control group (at the beginning of the treatment). Experiments complied with the current laws of France and were approved by Institut Curie ethical committee.

***Statistical analyses***

Statistical analyses were performed with Prism (version 5 and 8.3.1; GraphPad Software Inc.) and PASW Statistics (version 18.0; SPSS Inc.). ROC (receiver operating characteristic) analyses were performed to discriminate high and low Kindlin-1 and/or EGFR expressing groups. Survival distributions were estimated by the Kaplan Meier method.

***Data availability***

The data generated in this study are available within the article and its supplementary data files. Certain expression profile data from patients analyzed in this study were obtained from Gene Expression Omnibus (GEO) at GSE33072, GSE33072 and GSE5851. Microarray expression from the different cancer cell lines from the Cancer Cell Line Encyclopedia (CCLE) dataset and RNA-Seq from human tumors from the TCGA project and the OncoSG dataset that were analyzed in this study were obtained from cBioPortal ([www.cbioportal.org/](http://www.cbioportal.org/)).
